# Associations between EGFR gene polymorphisms and susceptibility to glioma: a systematic review and meta-analysis from GWAS and case-control studies

**DOI:** 10.18632/oncotarget.21011

**Published:** 2017-09-18

**Authors:** Xiao Yu, Nian Rong Sun, Hai Tao Jang, Shi Wen Guo, Min Xue Lian

**Affiliations:** ^1^ Department of Neurosurgery of The First Affiliated Hospital of Xi’an Jiaotong University School of Medicine, Xi’an, Shaanxi Province, China; ^2^ Department of Neurosurgery of Luonan County People's Hospital, Luonan County, Shaanxi Province, China

**Keywords:** epidermal growth factor receptor, glioma, polymorphism, meta-analysis

## Abstract

The results of genome-wide association studies (GWAS) and case-control studies performed to investigate the associations between epidermal growth factor receptor (EGFR) gene polymorphisms and glioma risk are controversial. The aim of this systematic review and meta-analysis is to determine whether EGFR gene polymorphisms are associated with glioma risk by searching ‘PubMed’, ‘EMBASE’, ‘Web of Science’, ‘Cochrane Library’ and ‘China WeiPu Library’ to retrieve studies that investigated associations between EGFR gene polymorphisms and glioma risk. Four GWAS containing 35 studies and 7 case-control studies meeting the inclusion criteria were finally recruited, and 11 single-nucleotide polymorphisms (SNPs) were analyzed. The results showed a significant positive association between rs730437/rs845552 and glioma risk in Asians, and a significant negative association between them in Caucasians. In addition, rs11506105 was significantly associated with an increased risk of glioma in both Asians and Caucasians, and rs11979158 decreased the risk of glioma in Caucasians. However, no significant association was observed between rs12718945/rs17172432/rs4947492 and glioma risk in Asians, between rs2252586 and glioma risk in Caucasians, and between rs3752651 and glioma risk in either Asians or Caucasians. In conclusion, different SNPs in EGFR gene might have different impacts on the risk of glioma in various ethnicities, which offers new insights into the treatment with a target-oriented approach.

## INTRODUCTION

Glioma is the most common primary brain tumor, with about 20000 new cases per year in the United States [1–3]. It has been verified that gliomas are correlated with a low survival rate, with a median overall survival (OS) of 8–15 months [4].

It is generally believed that glioma is very difficult to be cured because many currently available drugs that are effective against systemic cancers are unable to cross the blood-brain barrier [5]. To prevent and cure this devastating disease, it is therefore necessary to identify relative risk factors and the exact etiology of glioma, knowing that the etiology of glioma invloves several multiple factors [6–10] and and the actual pathogenesis remains unclear [11].

In recent years, more and more attentions have been paid to the roles of genetic factors (genes and gene polymorphisms) in the etiology, pathogenesis and complexity of glioma. Researchers attempt to explore new revolutionary therapeutic approaches based on these molecular genetics [1, 12]. Several gene polymorphisms, such as EGF-containing fibulin-like extracellular matrix protein 1 (EFEMP1) and cocaine-amphetamine-regulated transcript (CART) [13–15], have been identified to be significantly correlated with the etiology, development and progression of glioma.

Single-nucleotide polymorphisms (SNPs) in EGFR gene are getting increasingly recognition of importance in the etiology, development and progression of glioma, as it has been verified in many studies that EGFR gene plays a key role in human tumors by regulating cellular processes [10]. Wang et al. [10] reported that two polymorphisms (rs730437 and rs1468727) in EGFR gene were associated with etiology of giloma and its risk in Chinese populations, which was consistent with the study of Yan et al. [11]. Hou et al. [16] found EGFR haplotype ‘AATT’ was significantly associated with the decreased risk of glioma, while constrast results were observed in glioma patients carrying haplotype ‘CGTC’. In addition, genome-wide association studies (GWAS) [17–19] have also been performed and the results suggested that SNPs at two loci of EGFR gene (rs11979158 and rs2252586) were associated with glioma risk. Although an increasing number of EGFR gene polymorphisms have been identified to play an important role in etiology of glioma and be significantly associated with glioma risk [10, 11, 16–24], no comprehensive study has been performed to detect the associations between these EGFR gene polymorphisms and glioma risk, and give us conclusive results. The objective of this systematic review and meta-analysis is to determinate the associations of EGFR gene polymorphisms with glioma risk based on GWAS and case-control studies.

## MATERIALS AND METHODS

### Literature search

A systematic online search was conducted to find out all the eligibile studies. Databases including ‘PubMed’, ‘EMBASE’, ‘Web of Science’, ‘Cochrane Library’ and ‘China WeiPu Library’ were searched. All the studies recruited in our meta-analysis should investigate associations between EGFR gene polymorphisms and glioma risk. We used the following search terms to identify all the relevant studies: (‘EGFR’ OR ‘epidermal growth factor receptor’) AND (‘glioma’) AND (‘SNP’ OR ‘polymorphism’ OR ‘single nucleotide polymorphism’ OR ‘variation’). There were no language restrictions in our searching procedure. The reference lists of the recruited studies, reviews or conference reports were also searched to find out other eligible studies that might be omitted in databases. Furthermore, we examined the reviews and comments to further search out eligible studies so that no possible studies would be missed.

### Inclusion and exclusion criteria

The inclusion criteria of were as follows: (1) case-control studies that explored the relationships between EGFR gene polymorphisms and glioma risk or GWAS; (2) cases: patients diagnosed as glioma; controls: cancer-free subjects (Hospital-based or Healthy-Based); (3) evaluation of glioma risk and at least one EGFR gene polymorphism was analyzed and reported; (4) reporting detailed numbers or frequencies of alleles or genotypes in at least one EGFR gene polymorphism in both cases and controls subjects. The exclusion criteria were as follows: (1) case reports or reviews; (2) no currently available or sufficient data; (3) duplicated studies.

### Data extraction

Two authors extracted the general information and data in each study. If there was any disagreement, a third author would extract them, which was solved by consensus. The following data were collected: authors, publication year, types of article, number of studies in eligibile references, population ethnicities, sample size, source of controls, Hardy-Weinberg equilibrium (HWE) and SNPs studied in articles. We would contact the corresponding authors if there were incomplete data in recruited studies. In addition, whether the genotype distributions followed the HWE in controls was also explored.

### Data synthesis and statistical analysis

We calculated the odds ratio (OR) and 95% confidence interval (CI) to evaluate the associations between EGFR gene polymorphisms and glioma risk. We used allele contrast model to assess the main results of the strength of associations between EGFR gene polymorphisms and glioma susceptibility. Other genetic models were also calculated to evaluate the relationships between EGFR gene polymorphisms and glioma risk. We examined the heterogeneity of included studies using Q statistical test and I^2^ metric value. If I^2^ value was > 50% or *P* < 0.10, OR was pooled by random effects models; otherwise, OR was pooled by the fixed effects model. We also assessed the HWE for the control group in each article; a *P* value > 0.05 suggested that the controls followed a HWE balance. Furthermore, we also performed a sensitivity analysis to detect whether each study has a great impact on pooled results. Besides, subgroup analysis was also performed according to the ethnicity. Stata 14.0 software was used to calculate the pooled results, and a *P <* 0.05 was considered as statistically significant.

## RESULTS

### Study selection and characteristics

**Figure 1:**
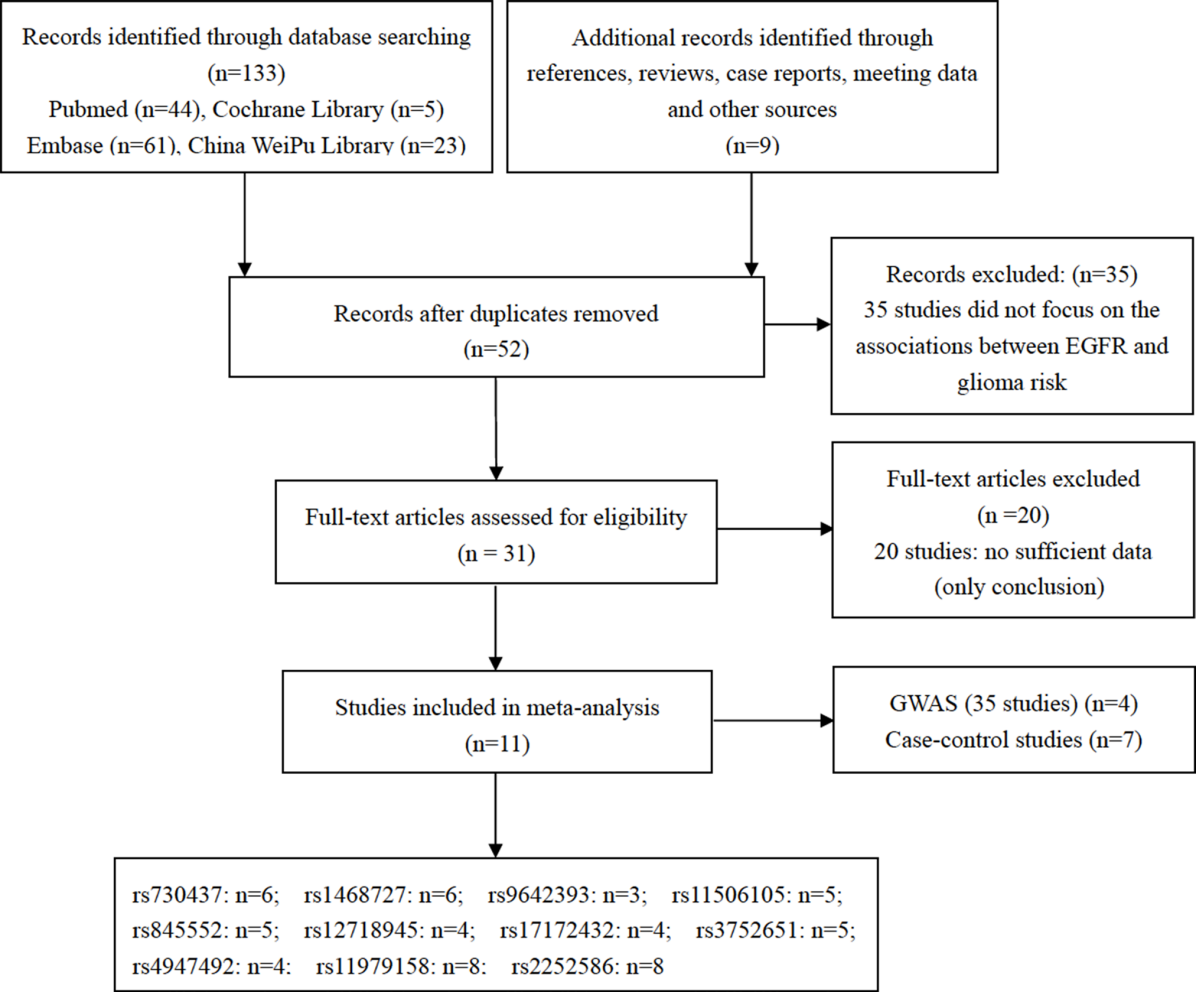
Flow chart showing the process of selection

A total of 11 studies [10, 11, 16–24] including four GWAS [17–19, 21] and seven case-control studies [10, 11, 16, 20, 22–24] were finally recruited in our study, as shown in Figure 1. GWAS by Rajaraman et al. [18], Di Stefano et al. [17], Melin et al. [21], and Sanson et al. [19] included 18, 7, 6 and 4 studies, respectively. Five studies [10, 11, 16, 20, 24] were performed in Asian populations, and the other six studies [17–19, 21–23] were conducted in Caucasian populations. Alleles and genotypes of 11 SNPs were finally collected and analyzed in our study, including rs730437, rs1468727, rs11506105, rs845552, rs12718945, rs17172432, rs3752651, rs4947492, rs9642393, rs11979158 and rs2252586. Four studies [11, 16, 20, 24] focused on the association between EGFR rs730437, rs1468727, rs11506105, rs845552, rs12718945, rs17172432, rs3752651, rs4947492 polymorphisms and glioma risk, among which two studies [16, 20] also reported the alleles and genotypes of EGFR rs9642393 in patients with glioma and controls. Wang et al. [10] studied the relationships between EGFR rs730437 and rs1468727 polymorphisms and glioma risk, and Andersson et al.’s study [22] explored the relationships between EGFR rs730437, rs1468727, rs9642393, rs11506105, rs845552, rs3752651 polymorphisms and glioma risk. In addition, four GWAS [17–19, 21] and one nested case-control study [23] were searched out and recruited in the pooled analysis of associations between EGFR rs11979158 and rs2252586 polymorphisms and glioma susceptibility. Controls in two studies were hospital-based [19, 20], and the remaining nine studies were population-based [10, 11, 16–18, 21–24]. All the controls in these studies complied with HWE. The general characteristics of recurited studies were shown in Table 1.

**Table 1:** Characteristics of studies selected in the meta-analysis

Author	Year	Type of article	Number of studies in article	Ethnicity	Sample Size		Source of controls	HWE	SNPs
					Case	Control			
Yan et al. [11]	2017	Case-control study	-	Asian	394	298	Population-Based	0.87	rs730437, rs1468727, rs11506105, rs845552, rs12718945, rs17172432, rs3752651, rs4947492
Wang et al. [10]	2015	Case-control study	-	Asian	300	300	Population-Based	0.78	rs730437, rs1468727
Du et al. [24]	2015	Case-control study	-	Asian	423	302	Population-Based	0.52	rs730437, rs1468727, rs11506105, rs845552, rs12718945, rs17172432, rs3752651, rs4947492
Wibom et al. [23]	2015	Nested case–control study	-	Caucasian	598	595	Population-Based	0.34	rs11979158, rs2252586
Rajaraman et al. [18]	2013	GWAS	18	Caucasian	1856	4955	Population-Based	0.27	rs11979158, rs2252586
Stefano et al. [17]	2013	GWAS	7	Caucasian	1372	1190	Population-Based	0.10	rs11979158, rs2252586
Melin et al. [21]	2013	GWAS	6	Caucasian	1431	2868	Population-Based	0.33	rs11979158, rs2252586
Jin et al. [20]	2013	Case-control study	-	Asian	72	302	Hospital-Based	0.46	rs730437, rs1468727, rs9642393, rs11506105 rs845552, rs12718945, rs17172432, rs3752651, rs4947492
Hou et al. [16]	2012	Case-control study	-	Asian	301	302	Population-Based	0.95	rs730437, rs1468727, rs9642393, rs11506105, rs845552, rs12718945, rs17172432, rs3752651, rs4947492
Sanson et al. [19]	2011	GWAS	4	Caucasian	6416	9935	Hospital-Based	0.07	rs11979158, rs2252586
Andersson et al. [22]	2010	Case-control study	-	Caucasian	725	1610	Population-Based	0.74	rs730437, rs1468727, rs9642393, rs11506105, rs845552, rs3752651

### Meta-analysis results

rs730437 was significantly associated with the increased risk of glioma (CC *vs.* CA/AA: OR = 1.38, 95% CI = 1.18–1.61, *P* < 0.001) in the overall populations, as shown in Table 3. Our subgroup analysis showed that there was a significant association between EGFR rs730437 polymorphism and susceptibility to glioma in Asian populations (C vs. A: OR = 1.33, 95% CI = 1.19-1.48, *P* < 0.001; CA vs. AA: OR = 1.36, 95% CI = 1.15-1.62, *P* < 0.001; CC vs. AA: OR = 1.82, 95% CI = 1.44-2.31, *P* < 0.001; CC/CA vs. AA: OR = 1.47, 95% CI = 1.25-1.72, *P* < 0.001; CC vs. CA/AA: OR = 1.52, 95% CI = 1.23-1.89, *P* < 0.001), while there was no significant association between EGFR rs730437 polymorphism and glioma risk in Caucasians (CC *vs.* CA/AA: OR = 1.24, 95% CI = 0.99-1.54, *P* = 0.058), as indicated in Table 2 and Table 3.

**Table 2:** Main results of meta-analysis of EGFR gene polymorphisms and risk of glioma

SNP	Major/Minor	Ethnicity	MAF		Test of association		Model	Test of heterogeneity	
			Cases	Controls	OR (95% CI)	*P* value		I^2^	*P* value
rs730437	A/C	Asian	0.43	0.36	**1.33 (1.19–1.48)**	**< 0.001**	F	0.0	0.874
rs1468727	T/C	Asian	0.49	0.42	**1.30 (1.17–1.45)**	**< 0.001**	F	0.0	0.851
rs9642393	C/T	Asian	0.43	0.38	**1.24 (1.02–1.51)**	**0.030**	F	0.0	0.541
rs11506105	A/G	Asian	0.41	0.36	**1.23 (1.09–1.39)**	**0.001**	F	0.0	0.868
rs845552	G/A	Asian	0.41	0.36	**1.24 (1.10–1.40)**	**0.001**	F	0.0	0.997
rs12718945	G/T	Asian	0.35	0.36	0.94 (0.83–1.06)	0.316	F	28.9	0.239
rs17172432	T/C	Asian	0.10	0.11	0.89 (0.73–1.08)	0.229	F	0.0	1.000
rs3752651	T/C	Asian	0.08	0.07	1.12 (0.90–1.40)	0.310	F	0.0	1.000
rs4947492	A/G	Asian	0.36	0.35	1.03 (0.91–1.16)	0.661	F	0.0	0.804
rs11979158	A/G	Caucasian	0.14	0.18	**0.81 (0.76–0.86)**	**< 0.001**	F	12.9	0.329
rs2252586	G/A	Caucasian	0.32	0.29	1.10 (0.98–1.23)	0.092	R	82.0	< 0.001

MAF: Minor Allele Frequency.

R: Random Effect Model.

F: Fixed Effect Model.

**Table 3:** Associations between SNPs in EGFR gene and risk of glioma in other genetic models

Genetic Models	Ethnicity	Test of association			Model	Test of heterogeneity	
		OR	95% CI	*P* value		I^2^ (%)	*P* value
rs730437 A/C							
Heterozygote model (CA *vs.* AA)	Asian	1.36	1.15–1.62	< 0.001	F	0.0	0.961
Homozygote model (CC *vs.* AA)	Asian	1.82	1.44–2.31	< 0.001	F	0.0	0.981
Dominant model (CC/CA *vs.* AA)	Asian	1.47	1.25–1.72	< 0.001	F	0.0	0.950
Recessive model (CC *vs.* CA/AA)	Overall	1.38	1.18–1.61	< 0.001	F	0.0	0.779
	Asian	1.52	1.23–1.89	< 0.001	F	0.0	0.998
	Caucasian	1.24	0.99–1.54	0.058	F	-	-
rs1468727 T/C							
Heterozygote model (CT *vs.* TT)	Asian	1.15	0.96–1.38	0.124	F	0.0	0.810
Homozygote model (CC *vs.* TT)	Asian	1.81	1.44–2.28	< 0.001	F	0.0	0.994
Dominant model (CC/CT *vs.* TT)	Asian	1.32	1.11–1.56	0.002	F	0.0	0.875
Recessive model (CC *vs.* CT/TT)	Overall	1.34	0.91–2.00	0.142	R	78.8	0.001
	Asian	1.66	1.36–2.02	< 0.001	R	0.0	0.998
	Caucasian	0.55	0.34–0.87	0.010	R	-	-
rs9642393 C/T							
Heterozygote model (CT *vs.* CC)	Asian	1.35	0.79–2.30	0.274	R	58.6	0.120
Homozygote model (TT *vs.* CC)	Asian	1.55	1.02–2.33	0.039	F	0.0	0.729
Dominant model (TT/CT *vs.* CC)	Asian	1.32	0.99–1.77	0.057	F	43.5	0.184
Recessive model (TT *vs.* CT/CC)	Overall	1.00	0.57–1.75	0.999	R	74.4	0.020
	Asian	1.35	0.93–1.95	0.112	R	0.0	0.653
	Caucasian	0.64	0.43–0.94	0.022	R	-	-
rs11506105 A/G							
Heterozygote model (AG *vs.* AA)	Asian	1.24	1.02–1.50	0.032	F	0.0	0.945
Homozygote model (GG *vs.* AA)	Asian	1.58	1.20–2.09	0.001	F	0.0	0.997
Dominant model (GG/AG *vs.* AA)	Asian	1.31	1.09–1.57	0.004	F	0.0	0.959
Recessive model (GG *vs.* AG/AA)	Overall	1.35	1.13–1.60	0.001	F	0.0	0.975
	Asian	1.40	1.09–1.82	0.010	F	0.0	0.998
	Caucasian	1.29	1.02–1.64	0.035	F	-	-
rs845552 G/A							
Heterozygote model (AG *vs.* GG)	Asian	1.24	1.02–1.51	0.033	F	0.0	0.969
Homozygote model (AA *vs.* GG)	Asian	1.48	1.14–1.93	0.004	F	0.0	0.999
Dominant model (AA/AG *vs.* GG)	Asian	1.30	1.08–1.56	0.005	F	0.0	0.987
Recessive model (AA *vs.* AG/GG)	Overall	1.12	0.82–1.54	0.462	R	65.7	0.033
	Asian	1.32	1.04–1.69	0.025	R	0.0	0.986
	Caucasian	0.80	0.63–1.00	0.054	R	-	-
rs3752651 T/C							
Recessive model (CC *vs.* CT/TT)	Overall	1.34	0.81–2.23	0.252	F	0.0	0.741
	Asian	1.99	0.18–22.10	0.574	F	-	-
	Caucasian	1.32	0.78–2.21	0.298	F	-	-
rs11979158 A/G							
Heterozygote model (AG *vs.* AA)	Caucasian	0.81	0.74–0.88	< 0.001	F	29.8	0.223
Homozygote model (GG *vs.* AA)	Caucasian	0.71	0.55–0.91	0.006	F	0.0	0.643
Dominant model (GG/AG *vs.* AA)	Caucasian	0.80	0.74–0.87	< 0.001	F	24.1	0.261
Recessive model (GG *vs.* AG/AA)	Caucasian	0.75	0.58–0.96	0.022	F	0.0	0.623
rs2252586 G/A							
Heterozygote model (AG *vs.* GG)	Caucasian	1.08	0.91–1.28	0.395	R	79.1	0.001
Homozygote model (AA *vs.* GG)	Caucasian	1.21	0.89–1.65	0.231	R	83.7	< 0.001
Dominant model (AA/AG *vs.* GG)	Caucasian	1.10	0.90–1.34	0.342	R	86.0	< 0.001
Recessive model (AA *vs.* AG/GG)	Caucasian	1.17	0.93–1.47	0.171	R	72.2	0.006

rs1468727 was a risk contributor to glioma susceptibility in Asians (C vs. T: OR = 1.30, 95% CI = 1.17-1.45, *P* < 0.001; CC *vs.* TT: OR = 1.81, 95% CI = 1.44-2.28, *P* < 0.001; CC/CT *vs.* TT: OR = 1.32, 95% CI = 1.11-1.56, *P* = 0.002; CC *vs.* CT/TT: OR = 1.66, 95% CI = 1.36-2.02, *P* < 0.001), and a protective contributor in Caucasians (CC *vs.* CT/TT: OR = 0.55, 95% CI = 0.34-0.87, *P* = 0.010). However, there was no significant association between rs1468727 and glioma risk in the overall populations (CC *vs.* CT/TT: OR = 1.34, 95% CI = 0.91-2.00, *P* = 0.142). All the data are shown in Table 2 and Table 3.

As shown in Table 2 and Table 3, although no significant association was observed between rs9642393 and glioma risk in the overall populations (TT *vs.* CT/CC: OR = 1.00, 95% CI = 0.57-1.75, *P* = 0.999), rs9642393 might increase the risk of glioma in Asian populations (T *vs.* C: OR = 1.24, 95% CI = 1.02-1.51, *P* = 0.030; TT *vs.* CC: OR = 1.55, 95% CI = 1.02-2.33, *P* = 0.039), and decrease the risk of glioma in Caucasian populations (TT *vs.* CT/CC: OR = 0.64, 95% CI = 0.43-0.94, *P* = 0.022).

Our results also illustrated a significant association between rs11506105 and glioma risk in either overall populations (GG *vs.* AG/AA: OR = 1.35, 95% CI = 1.13-1.60, *P* = 0.001), Asian populations (G vs. A: OR = 1.23, 95% CI = 1.09-1.39, *P* = 0.001; AG *vs.* AA: OR = 1.24, 95% CI = 1.02-1.50, *P* = 0.032; GG *vs.* AA: OR = 1.58, 95% CI = 1.20-2.09, *P* = 0.001; GG/AG *vs.* AA: OR = 1.31, 95% CI = 1.09-1.57, *P* = 0.004; GG *vs.* AG/AA: OR = 1.40, 95% CI = 1.09-1.82, *P* = 0.010) or Caucasian populations (GG *vs.* AG/AA: OR = 1.29, 95% CI = 1.02-1.64, *P* = 0.035).

We also found that rs845552 was significantly associated with increased risk of glioma in Asian populations (A vs. G: OR = 1.24, 95% CI = 1.10-1.40, *P* = 0.001; AG *vs.* GG: OR = 1.24, 95% CI = 1.02-1.51, *P* = 0.033; AA *vs.* GG: OR = 1.48, 95% CI = 1.14-1.93, *P* = 0.004; AA/AG *vs.* GG: OR = 1.30, 95% CI = 1.08-1.56, *P* = 0.005; AA *vs.* AG/GG: OR = 1.32, 95% CI = 1.04-1.69, *P* = 0.025); while no significant association between rs845552 and glioma risk was observed in either Caucasian populations or overall populations (all, *P* > 0.05, Table 2 and Table 3).

rs11979158 was found to play a protective role in etiology and risk of glioma in Caucasians (G vs. A: OR = 0.81, 95% CI = 0.76-0.86, *P* < 0.001; AG *vs.* AA: OR = 0.81, 95% CI = 0.74-0.88, *P* < 0.001; GG *vs.* AA: OR = 0.71, 95% CI = 0.55-0.91, *P* = 0.006; GG/AG *vs.* AA: OR = 0.80, 95% CI = 0.74-0.87, *P* < 0.001; GG *vs.* AG/AA: OR = 0.75, 95% CI = 0.58-0.96, *P* = 0.022), although no study was performed to detect the relationship between rs11979158 and glioma risk in Asian and overall populations (Table 2 and Table 3).

We failed to find any significant association between rs12718945, rs17172432 and rs4947492 and glioma risk in Asian populations, and between rs2252586 and glioma risk in Caucasians (all, *P* > 0.05), as shown in Table 2 and Table 3. We could not find a definite relationships between rs12718945, rs17172432 and rs4947492 and glioma susceptibility in Caucasians, and between rs2252586 and giloma risk in Asians. In addition, rs3752651 was not significantly associated with glioma risk in either overall populations, Caucasians or Asians (all, *P* > 0.05). All the data were shown in Table 2 and Table 3.

### Sensitivity analysis and publication bias

Furthermore, we also performed the sensitivity analysis. Omission of any study did not affect the previous results, suggesting that our results were relatively stable. However, we did not conduct the publication bias because it was not suitable to assess publication bias when the number of included studies was relatively small.

## DISCUSSION

EGFR is a transmembrane tyrosine kinase in 7p11.2, which has been verified as an important contributor to multiple cellular processes, such as cell division, migration, adhesion, differentiation and apoptosis [19]. EGFR is one of the critical oncogenes for several cancers [25], such as non-small cell lung cancer (NSCLC) [26] and urinary bladder cancer (BC) [27].

EGFR is a highly variable gene, which is thought as the fourth most highly mutated gene in a compendium of common cancer genes [28]. Recently, researchers have realized the importance of EGFR gene polymorphims in cancer therapy. EGFR yrosine kinase inhibitors (TKIs) have been verified to be effective in treating EGFR-mutant NSCLC, which are also considered as first-line treatment options in these patients [29]. Railkar et al. [27] described a targeted form of photo-therapy called photoimmunotherapy (PIT) that targets EGFR-expressing BC in their study, and found that anti-EGFR antibody panitumumab (Pan) IR700-induced PIT selectively killed EGFR-expressing BC cells in vitro and in vivo, and therefore warrants further therapeutic study in orthotopic xenografts of BC and ultimately in patients.

Many tumors, including glioma, show high resistance to conventional chemotherapy and/or radiotherapy. Ghotme et al. [1] thought that these conventional therapies did not take into account the unique molecular features of different subtypes of glioma. In recent years, it is widely believed that molecular genetics provide new insights in new revolutionary therapeutic approaches instead of conventional treatments [1]. Therefore, the associations between EGFR gene polymorphisms and susceptibility to glioma in different ethnicities have been widely studies [10, 11, 16–24], aiming to find new insights into treatment with a target-oriented approach in glioma.

Yan et al. [11] successfully genotyped 8 tSNPs in EGFR and found some evidence of association at two SNPs (rs730437 and rs1468727) that played a key role in glioma risk, which was consistent with some other studies [10, 16, 20, 24]. However, inconsistent findings were reported in Liu et al.’s study [30], indicating that the C allele of rs730437 was associated with decreased risk of glioma. Different genetic backgrounds, insufficient sample size, genotyping techniques or different environments may all contribute to these controversial results. In our study, rs730437, rs1468727 and rs11506105 were found to be significantly associated with the increased risk of glioma in Asians. Although these results are inconsistent with Liu et al.’s study [30], they might be more creditable than their study because more studies have been found out, and data of these studies were collected and analyzed in our meta-analysis. Furthermore, our results also showed EGFR rs730437 was not significantly associated with glioma risk in Caucasians, while we observed controversial results in the overall populations, suggesting that different genetic backgrounds might have significant impacts on the role of rs730437 in the etiology of glioma. As only one study [22] was performed in Caucasians and recruited in our meta-analysis, we could not make a definite conclusion about the role of rs730437 in giloma risk. Therefore, more studies with large sample size should be conducted to investigate the association between rs730437 and glioma risk in Caucasian populations. What’s more, the combination of different original data in each included study might lead to the changes of distribution of each genotype in different ethnicities. Therefore, it is easily for us to understand why there are significant differences in the pooled results in different ethnicities. Interestingly, our results suggested that rs1468727 was significantly associated with decreased risk of glioma in Caucasians, which was contrary to the case in Asian populations. Our findings were consistent with Liu et al.’s study [30], in which they observed that the rs1468727 T allele was significantly correlated with decreased glioma risk. The exact mechanism about how rs1468727 plays a key role in EGFR function, and why it plays a different role even contrast role in glioma risk in various ethnicities remain unclear. Therefore, more studies should be performed to investigate the exact mechanism of rs1468727 in etiology of glioma.

rs9642393 is another SNP in EGFR. Jin et al. [20] found that SNP rs9642393 in EGFR was associated with increased risk of glioma, while Hou et al. [16] did not find any significant association. Our subgroup analysis also indicated that rs9642393 played a key role in etiology and risk of glioma. Our results were more credible than each individual study. The distribution of alleles and genotypes might be changed when we put these origional data together. In our opinion, the changed distribution of alleles and genotypes might be the most important resason for the difference between our study and Jin et al. and Hou et al.’s studies [16, 20]. In addition, we also observed that rs9642393 was a protective factor to the risk of glioma in Caucasians, which is consistent with the study of Andersson et al. [22]. Different genetic backgrounds might constitute an important contributor to the difference between Caucasians and Asians, while other factors such as sample size, measurement errors or genotyping technologies also should not be ignored.

According to allele G association analysis and other genetic models, rs11506105 might play a key role in glioma risk in both overall, Caucasian and Asian populations, which was consistent with Yan et al.’s study [11]. However, Andersson et al. [22] reported that the rs11506105 A allele was associated with increased risk of glioma in Caucasian populations. In our opinion, rs11506105 might have different influences on etiology and risk of glioma in different ethnicities. However, what we could not neglect is that some other factors such as the sample size in different studies, different genotyping techniques, and the relationships between environment factors and genetic polymorphisms might also play play an imoprtant role in glioma risk.

The association between SNP rs845552 and glioma risk was discussed in five studie [11, 16, 20, 22, 24], and our pooled analysis showed that rs845552 was significantly correlated with glioma risk in Asians; however, rs845552 might not be an important contributor to the risk of risk in either overall or Caucasian populations. Our results are inconsistent with some other studies [11, 16, 20, 24], in which no statistical association with aglioma risk was observed, suggesting that either there was no such an effect, or the small sample size after stratified analysis limited the statistical power [11]. Another important reason why controversial results were found in our study and theirs [11, 16, 20, 24] was the re-distribution of numbers or frequencies of alleles and genotypes in glioma patients and control subjects. Therefore, it may easily understand why a significant association was observed in pooled results. In addition, our study showed that rs845552 did not play a key role in glioma risk in Caucasians. The different results between Asians and Caucasians might result from the different genetic backgrounds, sample size, measurement errors, genotyping technologies and the relationships between gene polymorphisms and environmental factors. Therefore, whether rs845552 was associated with glioma risk remains elusive and needs further investigation. In addition, rs12718945, rs17172432, rs3752651 and rs4947492 might not be important contributors to the etiology, risk and progression of glioma.

Four GWAS [17–19, 21] containing 35 studies and one case-control study [23] were finally recruited in our mat-analysis to explore the associations between rs11979158 and rs2252586 and glioma risk. Previously, Sanson et al. [19] conducted GWAS in UK and US glioma patients. Their analysis identified rs11979158 and rs2252586 in EGFR gene played an important role in risk and development of glioma. Rajaraman et al. [18] and Stefano et al. [17] conducted new independent GWAS of glioma to explore the association between rs11979158 and rs2252586 and glioma risk, and their results were consistent with Sanson et al. [19] However, Melin et al. [21] and Wibom et al. [23] failed to find any significant association between rs11979158 and rs2252586 and glioma risk. Wibom et al. [23] thought that it might be attributable to the relatively limited statistical power of their study. We observed that rs11979158 was significantly associated with glioma risk, which is consistent with Wu et al.’s study [31]. Although the same result was observed in our and Wu et al.’s study, our study is more credible than theirs because other two studies [18, 23] were searched out and included in our meta-analysis. Our study provides new insights into mechanisms of pathogenesis, development or progression, indicating that rs11979158 might be a candidate locus of the target-oriented approach. However, we did not find any study performed in Asians. Whether rs11979158 was correlated with glioma risk in Asian populations needs further investigation, knowing that different genetic backgrounds might have great impacts on the role of EGFR gene polymorphisms in glioma etiology. Wu et al.’s study [31] showed that rs2252586 had significant impact on glioma risk, which was inconsistent with our findings. Compared with Wu et al.’s study [31], our study had a larger sample size and more statistical power to detect the association. Therefore, in our opinion, rs2252586 might not be a candidate target for new therapies.

More attention should be paid to the implications of our results in clinical practice. First, rs2252586 could be considered as a useful markerfor glioma screening in certain ethnicities as we have found that the significant association between rs2252586 and glioma risk in different ethnicitie. Second, our study indicated that glioma is a monogenic disorder, and it is most probably associated with multiple genes. Third, screening of these EGFR gene polymorphisms may be useful for early identification of risk groups so that treatment effectiveness could be improved, surgical related complications could be avoided. In addition, these significant findings could also be helpful to cut down surgical and conventional treatment costs in patients with glioma.

Our study is the first meta-analysis to determinate the roles of 11 SNPs of EGFR in etiology and risk of glioma, which could offer new insights into treatment with a target-oriented approach. Furthermore, four GWAS containing 35 studies and 7 case-control studies were recruited and analyzed, which could provide sufficient statistical power to explore thees associations. However, some limitations should also not be ignored. First, no study was performed to detect the association between two important EGFR SNPs (rs11979158 and rs2252586) and glioma risk in Asians. Therefore whether rs11979158 and rs2252586 may be contributors to glioma in Asians needs more investigations. Second, we did not explore the association between EGFR gene polymorphisms and different subtypes of glioma such as astrocytoma, oligoastrocytoma and GBM due to the insufficient original data. As the biological characteristics may vary in different subtypes, whether EGFR gene polymorphisms play different roles in different subtypes of gliomas needs further investigation. Third, some other EGFR gene polymorphisms such as rs7809394, rs10225877 and rs917881 were not analyzed in our study, because only one study reported the relationships between these polymorphisms and glioma risk. Therefore, more larger-scale studies with sufficient origional data should be further performed.

## CONCLUSIONS

Different SNPs in EGFR gene might have different impact on the risk of glioma in various ethnicities. These EGFR gene polymorphisms provide new insights in treating glioma.
